# Subcutaneous pulsatile glucocorticoid replacement therapy

**DOI:** 10.1111/cen.12470

**Published:** 2014-01-01

**Authors:** Georgina M Russell, Claire Durant, Alia Ataya, Chrysoula Papastathi, Ragini Bhake, Wolfram Woltersdorf, Stafford Lightman

**Affiliations:** * Henry Wellcome Laboratories for Integrative Neurosciences and Endocrinology, Dorothy Hodgkin Building, University of BristolBristol, UK; † School of experimental psychology, University of BristolBristol, UK; ‡ Department of Clinical Biochemistry, UHBristolBristol, UK

## Abstract

The glucocorticoid hormone cortisol is released in pulses resulting in a complex and dynamic ultradian rhythm of plasma cortisol that underlies the classical circadian rhythm. These oscillating levels are also seen at the level of tissues such as the brain and trigger pulses of gene activation and downstream signalling. Different patterns of glucocorticoid presentation (constant *vs* pulsatile) result not only in different patterns of gene regulation but also in different neuroendocrine and behavioural responses. Current ‘optimal’ glucocorticoid replacement therapy results in smooth hormone blood levels and does not replicate physiological pulsatile cortisol secretion. Validation of a novel portable pulsatile continuous subcutaneous delivery system in healthy volunteers under dexamethasone and metyrapone suppression. Pulsatile subcutaneous hydrocortisone more closely replicates physiological circadian and ultradian rhythmicity.

## Introduction

The hypothalamic–pituitary–adrenal (HPA) axis is characterized by a circadian rhythm underpinned by a dynamic ultradian rhythm comprising discrete pulses of adrenocorticotropic hormone (ACTH) and glucocorticoid secretion in all mammalian species studied. In man, deconvolution analysis has revealed secretory pulses of cortisol every 80–110 min,^1^–^4^ which are reflected in the plasma with cortisol peaks approximately every 3 h.^5^ This HPA activity is tightly controlled via a feedforward/feedback system with natural inbuilt delays via signalling within the pituitary and adrenal glands^6^ with ACTH pulsatility being absolutely critical for normal adrenal function.^7^ Individual pulses of glucocorticoid are transmitted to the tissues 8 and provide an oscillating signal at the cellular level.^9^ Pulses of corticosterone/cortisol induce a pulse of glucocorticoid receptor (GR) binding that then triggers cyclic GR-mediated transcriptional regulation or gene pulsing. This is seen in cultured cells and animal models.^9^ Indeed, the same dose of glucocorticoid (constant or pulsatile) results not only in different patterns of gene regulation but also in differing neuroendocrine and behavioural responses.^10^ This implies that differential responsiveness of central nervous system's stress reactivity is dependent upon the pattern of glucocorticoid presentation.

Cortisol is an anticipatory hormone, and its levels start to rise first thing in the morning prior to awakening. Therapeutically, we treat patients requiring glucocorticoid replacement therapy with two or three times daily oral replacement regimes in an attempt to mimic their circadian cortisol rhythm. This has certain limitations; patients must wake up with very low cortisol levels and then take their morning dose of hydrocortisone. Hence, they have a shifted curve with their morning peaks occurring after they wake.^11^,^12^ Additionally, standard oral replacement produces smooth hormone levels and fails to address the underlying pulsatile ultradian rhythm. Despite our best efforts to provide optimal therapy and our success in preventing deaths from severe corticosteroid deficiency, mortality rates stubbornly remain double that of the background population – a risk similar to smoking. This is predominantly due to an increased incidence of cancer, cardiovascular and infectious disease.^13^ Patients also report poor health-related quality of life especially mental and physical fatigue.^14^ Of these, morning fatigue is particularly challenging. Reports show patients receiving three rather than twice-daily hydrocortisone have higher quality of life suggesting the pattern of steroid replacement is important.^11^,^14^,^15^ To overcome the problems assumed to be related to the pattern of cortisol replacement, we have developed a pulsatile glucocorticoid delivery system that more closely replicates normal physiology.

## Materials and methods

Local ethical and institutional approval was obtained. The study was discussed with the Medicines and Healthcare Products Regulatory Authority, and formal approval was deemed unnecessary. The study was conducted according to the principles of Good Clinical Practice and the Declaration of Helsinki. Preliminary studies were carried out on 23 healthy volunteers (7 females, 16 males) including (i) dose ranging of individual pulses, (ii) testing for optimal frequency of pulses to ensure appropriate peak and nadir levels throughout the day and (iii) combination of three different pulse doses over the 24 h to reproduce normal circadian and ultradian rhythmicity.

### Infusion pump

A portable subcutaneous infusion pump (Crono P, CANE Applied Medical Technology Ltd, Cambridge, UK) containing 100 mg of hydrocortisone in 1 ml (efcortesol®; Soverign, Essex UK) was made up to 10 ml with 0·9% saline and preprogramed to deliver a high-, medium- and low-sized pulse of hydrocortisone. All pulses were delivered at a flow rate of 10 μl/s. The pump delivered hydrocortisone subcutaneously via a cannula (Medtronic quick-set®, Medtronic MiniMed, Northridge, CA, USA) inserted into the abdominal subcutaneous tissue.

### Blood sampling

A venous cannula was also inserted in the antecubital fossa of the arm for 10-minutely blood sampling via the human automated blood sampling system (HABS).^5^ Blood samples were taken for cortisol every 10 min and ACTH every hour. During the blood sampling period, a standardized routine was adhered to.

### Experimental protocol

Experiments 1 and 2 were performed to establish the dose range and frequency of individual pulses of hydrocortisone. 21 healthy volunteers (7 females, 14 males) underwent dexamethasone suppression. 1 mg of dexamethasone was taken at midnight at home; a further 1 mg was taken at 9 a.m. at the research unit. Volunteers received two doses of hydrocortisone at varying time intervals (dose range 0·3–4 mg) over a 7-h period. Blood samples were taken 09·30–16·30.

Experiment 3a. A healthy male volunteer underwent dexamethasone suppression. 1 mg of dexamethasone was taken at 08·00 at home on day 1. A further 1 mg of dexamethasone was taken at 12·00, 1 mg at 18·00, 0·5 mg at 24·00 and 0·5 mg at 07·00 the following morning (day 2) at the research unit. The volunteer received a pulse of hydrocortisone via the pump every 3 h, starting with a high dose of 400 μl (4 mg) at 12·00, 15·00 and 18·00, a medium dose of 230 μl (2·3 mg) at 21·00, 24·00 and 03·00 and a low dose of 50 μl (0·5 mg) at 06·00 and 09·00 to give a total daily dose of 19·9 mg of hydrocortisone. Blood sampling commenced at 12·00 and finished at 12·00 the following day.

Experiment 3b. In this study, endogenous cortisol synthesis was inhibited in a healthy male volunteer with metyrapone. A starting dose of 250 mg tds was used, increasing by 250 mg tds every 1–2 days to 750 mg tds over 5 days. Metyrapone was taken at meal times to increase compliance and decrease gastric side effects. Hydrocortisone replacement therapy was again administered via the infusion pump using a high dose of 400 μl (4 mg) at 03·00, 06·00 and 09·00, a medium dose of 230 μl (2·3 mg) at 12·00, 15·00 and 18·00 and a low dose of 50 μl (0·5 mg) at 21·00 and 00·00 to give a total daily dose of 19·9 mg of hydrocortisone. After being attached to the pump, the volunteer underwent his usual daily routines. The line and syringe were changed on day 3 according to manufacturer's guidelines. This was performed at a location convenient to the volunteer. On day 4, he attended the research unit blood sampling commenced at 15·00 and finished at 15·00 the following day.

### Assays

Cortisol samples were allowed to clot at room temperature prior to centrifugation, and serum was frozen at −80°C until assayed. Samples for ACTH were collected in chilled EDTA tubes and kept on ice until centrifugation at 4°C within 30 min. Plasma was stored at −80°C until assayed. Analysis was performed by the department of clinical biochemistry at the University Hospitals of Bristol NHS Foundation Trust (UHBristol) using an electrochemiluminescence immunoassay (Cobas®; Roche, Indianapolis, IN, USA). Cross-reactivity with 11-deoxycortisol was 4·1%.

## Results

**Figure 1:**
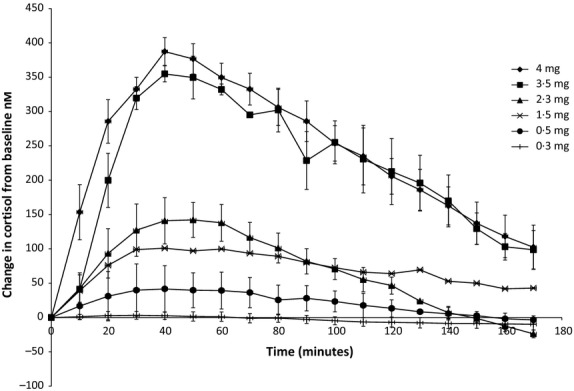
Plasma cortisol dose–response curves ± SEM to 4 mg (*n* = 8), 3·5 mg (*n* = 2), 2·3 mg (*n* = 4), 1·5 mg (*n* = 2), 0·5 mg (*n* = 4), 0·3 mg (*n* = 3) doses of subcutaneous hydrocortisone expressed as change in cortisol from baseline in dexamethasone-suppressed healthy volunteers.

**Figure 2:**
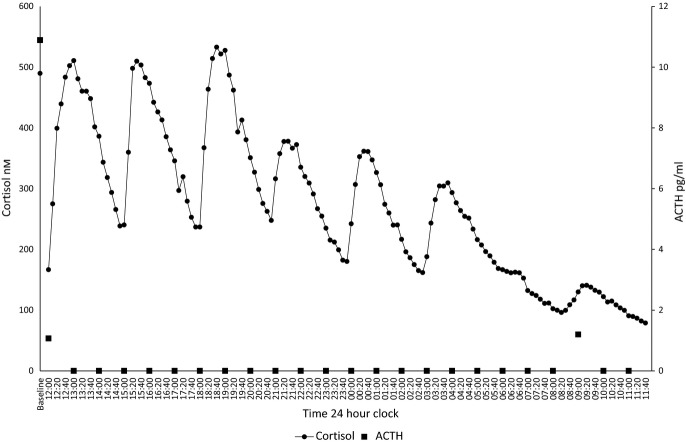
24-h cortisol (10 minutely) and ACTH (hourly) profiles in a dexamethasone-suppressed healthy male volunteer receiving hydrocortisone (total daily dose 19·9 mg) via a pulsatile portable subcutaneous infusion pump.

**Figure 3:**
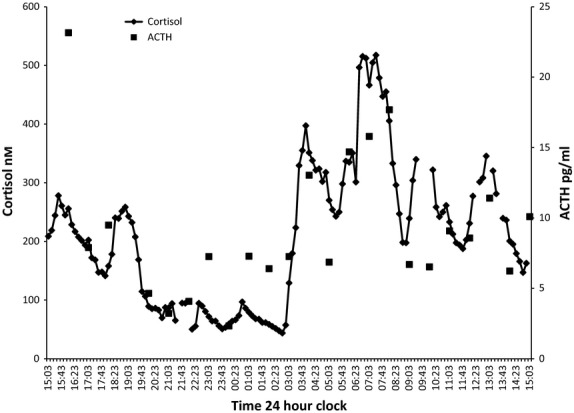
24-h cortisol (10 minutely) and ACTH (hourly) profiles in a healthy male volunteer following 5-day endogenous adrenal suppression with metyrapone whilst receiving hydrocortisone replacement therapy via a subcutaneous portable pulsatile infusion pump (total daily dose 19·9 mg).

Figure1 shows the dose response of plasma cortisol to different doses of subcutaneous cortisol in dexamethasone-suppressed volunteers. A high pulse size of 4 mg, medium 2·3 mg and low 0·5 mg was chosen. A pulse frequency of 3 h was chosen as at a higher frequency, there was an accumulation effect between pulses and the definitive on/off pattern of the pulse lost (data not shown). Fig.2 shows the 24 h profile of plasma cortisol utilizing our infusion paradigm under dexamethasone suppression (for practical reasons, timing of pulses was not synchronized to chronological time). A circadian peak of >500 nm and trough of <100 nm of cortisol were achieved; clear individual pulses of cortisol can be seen. This is combined with appropriate ACTH suppression confirming dexamethasone suppression of endogenous cortisol production. These results were confirmed in a separate study in which instead of dexamethasone inhibition of ACTH secretion, secretion of cortisol was suppressed with metyrapone. In this paradigm, our protocol of pulsatile hydrocortisone replacement therapy in fig.3 shows the 24 h profile of plasma cortisol. A circadian peak of >500 nm and a trough of <100 nm of cortisol were achieved. Clear individual pulses of cortisol can be seen. This is combined with appropriate ACTH concentrations confirming that despite inhibition of endogenous cortisol synthesis, our infused cortisol maintained normal physiological ACTH levels.

## Discussion

The HPA axis is characterized by a circadian peak of cortisol at about 09·00 of >500 nm and a trough at midnight of <100 nm.^1^Standard oral hydrocortisone replacement therapy usually splits the medication dose into three or even four times a day in an attempt to mimic the circadian pattern of release. This is clearly more advantageous than once/twice daily; however there is some evidence that increasing the frequency of oral replacement alters nadir levels.^16^ Plenadren® modified-release hydrocortisone,^17^ Chronocort®^18^ and constant subcutaneous hydrocortisone infusions^19^ have been developed to improve the circadian pattern of cortisol replacement. None of these regimens, however, take into account the true physiological pattern of cortisol secretion which occurs in individual pulses with a mathematically modelled mean interburst interval of 80–110 min 2–4 and plasma peaks and troughs approximately every 3 h.^5^ *In vitro* and *in vivo* work has shown that these pulses of glucocorticoid in the blood are also seen at the tissue level and induce a phenomenon known as gene pulsing.^20^ A pulse of glucocorticoid triggers a pulse of glucocorticoid receptor binding which then triggers a pulse of downstream activity. This is highly specific to natural (cortisol and corticosterone) as opposed to synthetic glucocorticoids.^9^ There is very clear evidence from animal models that oscillating levels of corticosteroid are vital for normal cognitive function.^10^ Indeed, the pattern of glucocorticoid exposure – and even the phase of the pulse – results in differential effects on behaviour and the c-fos response of the brain – particularly the amygdala – to a stressor. This is also likely to lead to metabolic and cardiovascular dysfunction.^21^–^23^ Current replacement regimes with their relatively constant glucocorticoid receptor occupancy and consequent abnormal glucocorticoid-dependent gene transcription undoubtedly contribute to glucocorticoid side effects and may even be a causal factor for the excessive morbidity and mortality of patients on glucocorticoid replacement.

The current report shows that it is possible to reproduce near physiological patterns of both circadian and ultradian rhythmicity using a specialized pump and simple subcutaneous infusion techniques. A cortisol circadian peak of 500 nm with a trough of <100 nm in combination with discrete pulses of cortisol was achieved, similar to healthy volunteer data using the same analytical technique.^5^,^24^ The pump was well tolerated by volunteers with no irritation or infections reported. Physiological cortisol production is approximately 8–12 mg/day whilst patients typically receive 20 mg a day.^25^,^26^ In this study, a standard patient dose was chosen as we needed proof of concept that it is possible to replicate a physiological pattern of replacement and safety had to be of foremost importance. We are now in a position not only to assess optimal dose but also to use this equipment in controlled clinical trials to assess the cognitive, metabolic, cardiovascular and immunological importance of normal physiological patterns of cortisol in patients on steroid replacement.
